# Pediatric obesity: prevention is better than care

**DOI:** 10.1186/s13052-020-00868-7

**Published:** 2020-07-24

**Authors:** Roberta Romanelli, Nicola Cecchi, Maria Grazia Carbone, Michele Dinardo, Giuseppina Gaudino, Emanuele Miraglia del Giudice, Giuseppina Rosaria Umano

**Affiliations:** 1 Clinical Nutrition Unit -Santobono-Pausilipon Children’s Hospital, Naples, Italy; 2 Department of the Woman, of the Child, of General and Specialized Surgery, University of Campania “Luigi Vanvitelli”, Via L. de Crecchio, 2, 80138 Naples, Italy

**Keywords:** Childhood obesity, Obesity prevention intervention, Family-based intervention, School-based intervention, Public health intervention

## Abstract

Pediatric obesity is one of the most relevant health issues of the last century. Obesity-related short and long-term consequences are responsible of a large amount of economic cost. In addition, the different therapeutic strategies, such as lifestyle correction, drug, and bariatric surgery have displayed low effectiveness. Considering this evidence, prevention appears to be more promising than treatment in contrasting obesity epidemic. In this review, we summarize obesity pathogenesis with the aim of highlight the main obesity risk factors that can be addressed as target of preventive interventions. Moreover, we report the evidence about effectiveness of different interventions targeting family, school, and community. A multiple-component intervention, addressing different targets and settings, might be desirable, however more studies are needed to confirm long-term efficacy and to direct policy interventions.

## Introduction

World Health Organization describes pediatric obesity epidemic as “one of the most serious public health challenges of the 21^st^ century” [1]*.* Recent estimates from WHO revealed that obesity prevalence has increased three times from 1975 to 2016 [2] and that about 41 million children aged under 5 years and 340 million children and adolescents aged 5–19 years are affected by overweight or obesity [3]. In Italy, about 21.3 and 9.3% of school-aged children are overweight and obese, respectively [4].

Obesity-related health costs derive also from its short and long-term comorbidities. Firstly, obese children are at higher risk of glucose intolerance [5], non-alcoholic fatty liver disease [6], dyslipidemia [7], and hypertension [8]. In addition, childhood obesity tends to persist during adulthood. It has been estimated that about 80% of severe obese children at the age of 2 years will be obese during adulthood [9]. Moreover, several evidence link childhood obesity to the burden of Non-Communicable Diseases in adult population [10–14]. Obesity is responsible for increased risk of type 2 diabetes [10], cardiovascular disease [11, 12], finally leading to increased morbidity and mortality [13, 14].

Despite scientific and clinical efforts, current therapies, including education, diet, exercise, drug, and bariatric surgery, to reduce obesity are failing to provide effective long-term results [15].

In view of this evidence, there is a strong need to identify the major obesity risk factors and to promote effective interventions to prevent obesity epidemic and its long-term consequences. In this review, we summarize the current knowledge about childhood obesity risk factors and prevention studies.

### Obesity pathogenesis

Obesity is a complex disease and the chronic mismatch between caloric intake and expenditure has been recognized as the main mechanism of weight gain [16]*.* Western diet and commercial interests that induce the consumption of cheap, energy-dense, and low-in-nutrients foods and beverages and the concurrent sedentary lifestyle are the main drivers of the obesity epidemic [17]. In addition, social inequalities enhance the risk of obesity being a low socioeconomic status associated with higher prevalence of obesity [18, 19]. However, not all the subjects exposed to an obesogenic environment develop obesity, therefore, over the past decades, genetic basis of obesity have been investigated.

#### The role of genetics

Genome wide association studies (GWASs) have reported the association between several single nucleotide polymorphisms (SNPs) and weight gain [20, 21]. Multiple gene variants might predispose subjects to gain weight in an obesogenic environment, this genetic form is referred as polygenic obesity [22, 23]. In addition to polygenic obesity, monogenic forms of obesity have been described. Single gene variations in the leptin-melanocortin hypothalamic pathway are responsible of severe early-onset obesity that are characterized by hyperphagia, rapid weight gain, and other possible endocrine dysfunctions [24–29]. However, monogenic obesities are responsible for a small percentage of obesity cases and cannot explain the obesity epidemic even if they increase the susceptibility to an obesogenic environment [30].

#### The role of epigenetics

During the last years, a great interest in the interaction between genes and environment has pointed out the role of epigenetics in obesity pathogenesis. Environmental factors can affect gene expression and activity influencing both transcription, translation, and post-translational processes without changing DNA sequence [22]. DNA transcription can be regulated by DNA methylation that inhibits the binding of transcriptional factors to gene promoter. In addition, histones acetylation can interfere with gene expression by changing chromatin structure. Finally, long non-coding RNA (lncRNA) and microRNA (miRNA) can affect RNA messenger levels and proteins production [31]. DNA methylation is the most investigated epigenetic change.

Epigenetic modifications are tissue-specific and might persist over time [32]. Intrauterine life and the first 2 years of life have been described as a crucial period for metabolic programming as the epigenome processes are more active and pliant [32]. Robust evidences have pointed out the association between maternal weight gain, health conditions, and tobacco smoke with offspring obesity [31, 33]. One of the most important examples of the transgenerational transmission of obesity risk is the Dutch Hunger Winter of 1944. Children born around the Dutch famine that were exposed to maternal undernutrition and showed higher prevalence of obesity, glucose intolerance, and cardiovascular disease later in life [34]. Similarly, maternal obesity and the consequent elevation of glucose, fatty acids, and amino acids plasmatic levels can influence the development and regulation of appetite, neuroendocrine function, and energy balance promoting obesity [35].

Considering this evidence, the characterization of epigenetic changes and their role in childhood obesity might enable the identification of early markers of risk, therapeutic targets, and prevention strategies.

#### The role of microbiota

Human gut hosts a complex and large bacterial community called microbiota. The composition of gut microbiota depends on diet composition, as different substrates can promote or inhibit the growth of different phyla. For example, high intake of fiber and fermented food promotes a healthier and more diversified microbiota. Conversely, western diet is associated with an unhealthy composition of microbiota [36]. Bacteria colonization of human intestine begins with delivery and continues during early nutrition stages [37]. Therefore, it is essential to care nutrition during neonatal period and first years of life. In particular, breastfeeding has been associated with a healthier composition of microbiota compared to infant formula. In fact, maternal milk is able to transfer several phyla, such as *Bifidobacterium*, *Streptococcus*, and *Lactobacillus*, that can contribute to neonatal microbiota [37]. Animal and human studies have associated gut dysbiosis with obesity in adult and children with contrasting results [38, 39]. Composition of gut microbiota differs between obese and lean subjects [36]. In particular, obese subjects have a higher colonization by *Firmicutes* and reduced levels of *Bacteroides* and *Prevotella* and weight loss has been associated with a reconstitution of *Bacteroides-Firmicutes* ratio as in lean subjects [40]. The proposed pathogenesis underpinning gut dysbiosis-obesity relationship include several mechanisms, such as alteration of gut layer, modulation of immune system and inflammation, production of metabolites able to target other organs including adipose tissue and liver. In particular, certain bacterial groups produces short chain fatty acids (SCFAs) from indigestible fibers. SCFAs serve as energy source for colonocytes as well as are able to reach other tissues inducing gluconeogenesis, de novo lipogenesis, epigenetic modifications, and incretin secretion [36]. Nevertheless, the complexity of obesity pathogenesis does not allow to demonstrate a clear and independent role of gut microbiota in obesity even though evidence has been provided.

### Obesity prevention

With the exception of genetic predisposition to obesity, it is clear that there are different modifiable targets for preventing childhood obesity. Additionally, a multicomponent approach, including political, social, and educational programs, should be promoted. Moreover, a multi-level intervention starting from the child, to family, school, and community may be applied (Table 1). Tailoring the type of intervention to children age (Table 2).

**Table 1:** Obesity prevention interventions according to setting

Setting	Intervention
Family	Parents weight control
	Nutritional education
	Emotive eating education
	Lifestyle education
School	Provision of healthy foods
	Nutritional education
	Healthy environment with availability of healthy food and water
	Allow physical activity
	Physical activity education
Community	Education
	Taxation for unhealthy foods and beverages
	Industries incentives for heathy food and beverages
	Control of school meal
	Open free space for physical activity in urban areas
	Incentives for rural areas and low income families
	Incentives for free counselling

**Table 2:** Preventive intervention according to children age

Age	Intervention
Birth-2 years	Promote exclusive breastfeeding
	Controlled protein intake
	Avoid sugar sweetened beverages
	Promote variety of foods
	Avoid restrictive feeding habits
	Avoid screen time
2–12 years	Promote education and food knowledge
	Avoid sugar sweetened beverages
	Five meals a day
	60 min of physical activity daily
	Reduce screen time
	Five portions of fruit and vegetables daily
13–18 years	Allow child to choose the snack
	60 min of physical activity daily
	Teach how to plan and prepare meals

#### Family based interventions

Given the central role of parents in controlling and influencing children’s dietary habits, physical activity, and sleep, family-based prevention programs are promising tool in limiting obesity epidemic. As mentioned above, the early stages of life, from conception to 2 years of age, are associated with the risk of later obesity [41]. Parents weight control before conception and during pregnancy as well as tobacco smoke avoidance should be encouraged [41]. Therefore, educational sessions focusing on healthy diet, active lifestyle, and weight monitoring during pregnancy should be performed. Several evidences have pointed out the protective action of breastfeeding against obesity compared to formula feeding [42, 43]. In addition, excessive protein intake should be avoided during the first year of life [44–47]. Consequently, exclusive breastfeeding until 6 months and controlled protein intake after weaning should be encouraged [48, 49].

Several studies have investigated the effectiveness of prevention programs focused on the first 1000 days of life on weight gain with mixed findings. In particular, limited effect has been observed in anthropometric outcomes, but several behavioral improvements in terms of diet, lifestyle, and sleeping habits have been reported. The heterogeneity of the results can be attributed to the differences in intervention type and limited number of studies [50–52]. Therefore, more research is needed to recommend specific prevention programs.

Family dynamics are crucial in later ages too. During pre-school age, it is essential to promote healthy eating behaviors. Consequently, mealtime has a great importance as parents act as role models, educators, and health promoters of their children. Parents should offer a good variety of foods, in favor of fruits and vegetables, and should limit the consumption of sugar sweetened beverages [53, 54]. In addition, parents should take care of feeding practice during meals, offering a positive models in accepting new and healthy foods inducing imitation in the child [55]. A recent metanalysis reported that several parents’ educational practices such as verbal praise, modeling, availability, and active were significantly associated with child healthy eating behavior. Conversely, pressuring children to eat, rewarding food consumption, and restrictive guidance had a negative association with healthy food consumption [53].

Paralleling with nutrition, family education practice should reduce mass media exposure as they expose the child to junk high-calories foods [56]. In addition, television viewing and use of tablets and videogames increase sedentary behavior and reduce the time spent in active games and sports. Therefore, screen time is associated with a significant increase of the risk of being overweight or obese [57].

Family-based intervention programs have been associated with a significant improvement in adiposity measures and cardiometabolic risk profile. Moreover, children in the intervention group displayed a healthier eating pattern compared to control group [58–61].

#### School-based interventions

Schools have a pivotal role in children education, therefore they figure among the most relevant settings in childhood obesity prevention. Schools provide education, meals, and can promote healthy lifestyle.

A body of literature reports that students benefit of healthier school environment [62–67]. Intervention programs differ in terms of length, type of intervention (i.e. nutrition and/or physical activity), and target (i.e. students and/or families), however, they have promising results. Among the targets, reduction of sugar sweetened beverages has been evaluated. A cost-effectiveness analysis based on installation of free water dispensers on school lunch lines showed a significant reduction of economic costs through reduction of childhood obesity [64]. Other intervention programs have focused on eating education and knowledge promotion. The intervention group showed BMI reduction and lower consumption of unhealthy food and sugar sweetened beverages [63, 68]. Similarly, prevention via implementation of physical activity in school settings reported significant improvement in terms of adiposity measures and healthy behavioral changes [69].

In conclusion, current literature suggests that obesity prevalence can be slightly lowered through school interventions with a multicomponent approach (nutrition and physical activity). The wide heterogeneity of the studies may limit the exact identification of a well-defined program. Further research is needed to investigate the efficacy and long-term outcomes of these interventions.

#### Public policy interventions

Although family and school play a key role in children education, children are influenced by a broader social environment. Therefore, community prevention programs should be encouraged. Several different target have been addressed, such as sugar sweetened beverages consumption, social inequalities, urbanization, equitable access to services, positive events, and use of social network [70]. In particular, urbanization is one of the causes of childhood obesity as it has been associated with higher consumption of junk food and sedentary behavior [71]. In fact, the most ranked recommendations for urban communities are the implementation of the public open spaces and the reduction of sport facilities costs [72]. Nevertheless, at the same time urban environment enables a more feasible access to community prevention programs [73, 74]. In fact, children living in rural areas have a higher risk of obesity than urban counterpart [75]. A systematic review including studies on rural communities has reported effectiveness of policy intervention enhancing opportunity of outside physical activity [76].

During the last years, the reduction of sugar intake has been addressed. Several international societies recommend against the assumption of sugary drinks especially during the first years of life [77]. Therefore, a body of intervention against the consumption of sugar sweetened beverages has been promoted. In particular, several national policy have undertaken taxation of sugary drinks [78–83]. A systematic review reported that for each 10% increase in sugary drinks price with a tax it is estimated a reduced sugary drink consumption by 7% [84]. Sugary drinks taxation appears as one of the most promising prevention programs according to modeling studies and it can be of more impact if associated with other interventions enabling access to healthy foods [85, 86]. Another approach is the use of low-calorie sweeteners that contain no or few added sugars that have been associated with contrasting results [87].

While there is promising evidence of community based single intervention efficacy, there are limited data and knowledge for the multiple level interventions and how to combine the different interventions for obesity prevention.

## Conclusions

Prevention appears the most promising tool to counteract obesity epidemic. Education toward healthy nutrition and active lifestyle constitutes the basis of every type of intervention program. A multicomponent and multilevel approach involving community, school, and family might be more effective than single component programs. However, more research is needed to characterize and standardize the most effective protocol in terms of feasibility, sustainability, cost-effectiveness, and long-term outcomes.

## Data Availability

Not applicable.
